# Norcorrole as a Delocalized, Antiaromatic System

**DOI:** 10.1038/s41598-019-39972-y

**Published:** 2019-03-19

**Authors:** Jeanet Conradie, Cina Foroutan-Nejad, Abhik Ghosh

**Affiliations:** 1 Department of Chemistry, UiT – The Arctic University of Norway, 9037 Tromsø, Norway; 2 Department of Chemistry, University of the Free State, 9300 Bloemfontein, Republic of South Africa; 3 CEITEC – Central European Institute of Technology, Masaryk University, Kamenice 5, CZ 62500 Brno, Czech Republic

## Abstract

Nickel norcorrole provides an unusual example of a molecule that is strongly antiaromatic according to the magnetic criterion, but which exhibits, according to high-quality DFT calculations, a symmetric, delocalized structure with no difference in bond length between adjacent C_*meso*_-C_*α*_ bonds. A fragment molecular orbital analysis suggests that these discordant observations are a manifestation of the high stability of the dipyrrin fragments, which retain their electronic and structural integrity even as part of the norcorrole ring system.

## Introduction

**Figure 1:**
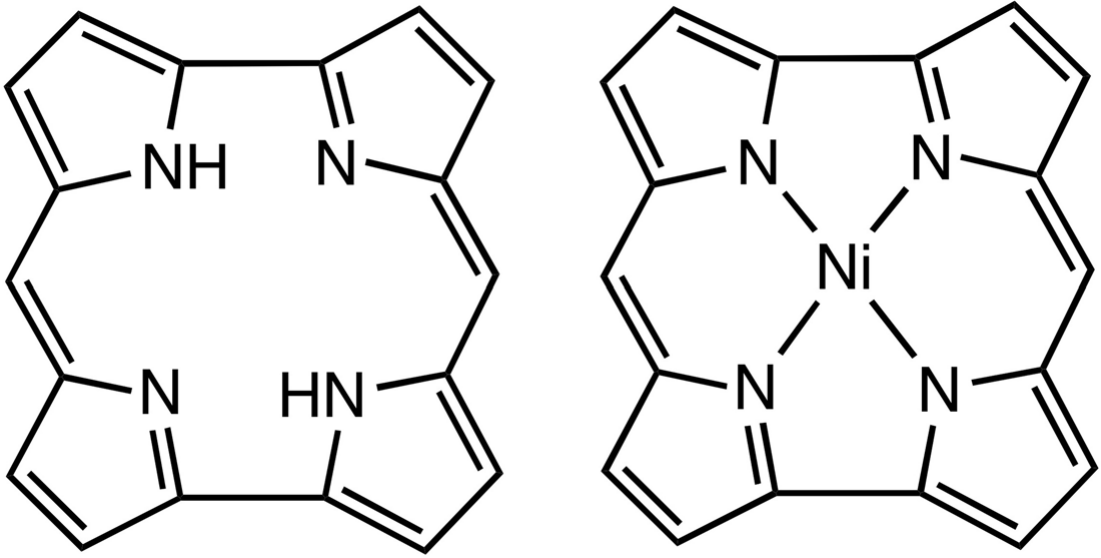
Free-base (H_2_Nc, left) and nickel norcorrole (NiNc, right).

Over a dozen years ago, one of us conceptualized norcorrole (H_2_Nc) as the smallest, realistic, fully conjugated tetrapyrrole ring system as part of a theoretical exercise (Fig. 1)^1^. Among the more notable conclusions of the study, which employed standard density functional theory (DFT) methods, was that nickel norcorrole (NiNc) should exhibit a slightly domed, but otherwise fully symmetric structure. In other words, the calculations did not indicate any difference in bond length between adjacent C_*meso*_-C_*α*_ bonds, as is typically observed for antiaromatic porphyrinoids^2–4^. Within a few years thereafter, norcorrole was experimentally realized by Bröring and coworkers in the form of an iron(III)-iodido complex^5^. Subsequently, Kobayashi, Shinokubo, and their coworkers reported a gram-scale synthesis of a nickel *meso*-diarylnorcorrole, clearing the path for wide-ranging investigations of the norcorrole derivatives^6^. Interestingly, the X-ray structure reported by these authors (CCDC: YEQKUC) revealed a planar macrocycle geometry with significant bond length alternation^6^. Furthermore, the experimental ^1^H NMR spectra and nucleus independent chemical shift (NICS) calculations clearly implicated NiNc as an antiaromatic system^6^. Together, these findings pose an interesting conundrum: although NiNc is antiaromatic according to the magnetic criterion, DFT geometry optimizations indicate a symmetric structure with little or no bond length alternation. While chemical theory does not rule out such a system, delocalized, antiaromatic systems are virtually unknown among real molecules. Presented herein is a detailed DFT investigation aimed at establishing the true equilibrium geometry of NiNc.

## Results and Discussion

**Figure 2:**
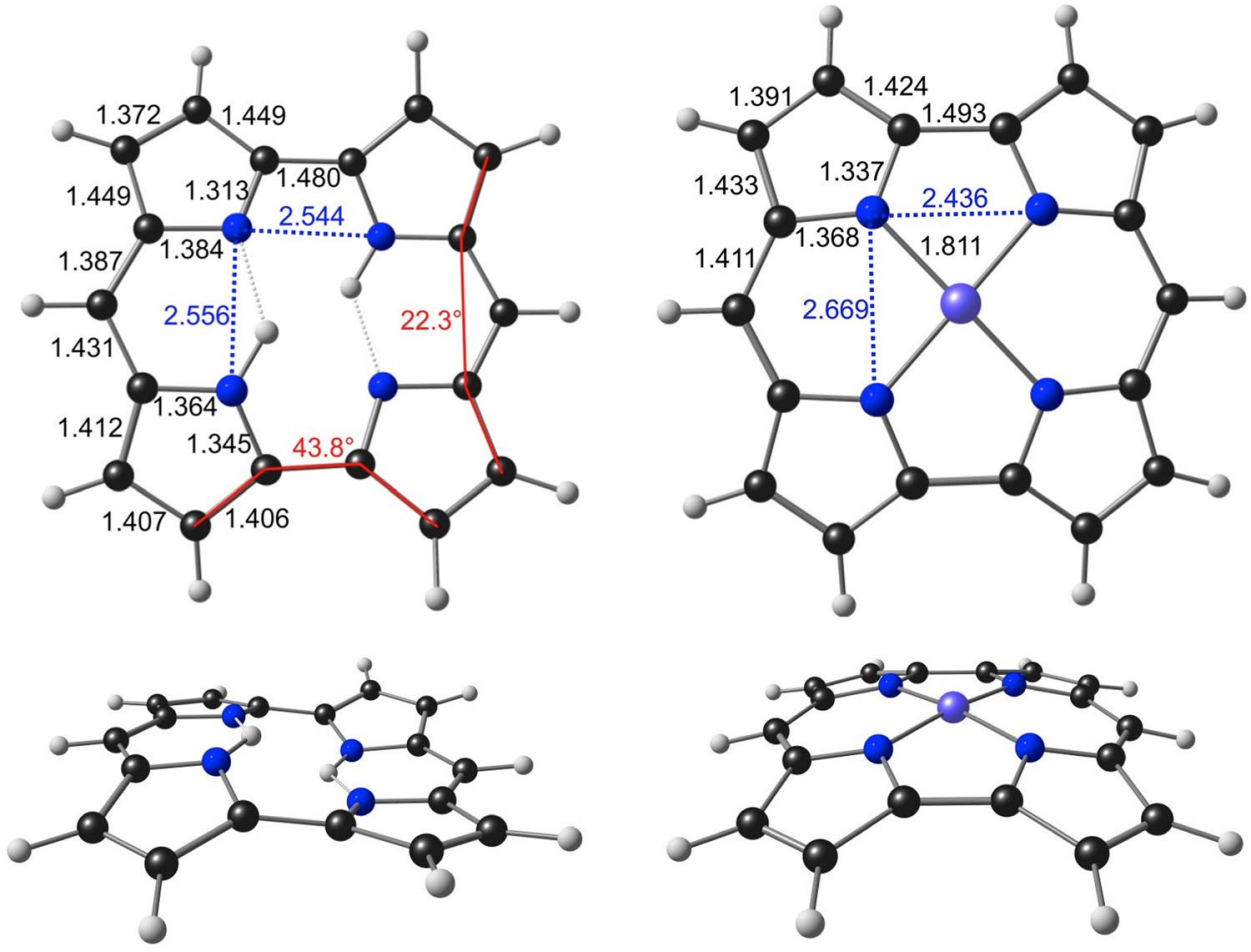
Selected B3LYP-D3/STO-TZ2P geometry parameters (Å, deg) of H_2_Nc (left) and NiNc (right).

Geometry optimizations with a variety of exchange-correlation functionals reproduced *all* key geometrical features noted in our earlier study^1^. Figure 2 depicts highlights of the B3LYP^7–9^ -D3^10^/STO-TZ2P optimized geometries. The energy minima turned out to be a *C*_i_-symmetric wave conformation for H_2_Nc^11^ and a *C*_2v_-symmetric dome conformation for NiNc. The planar *D*_2h_ form of NiNc, just 0.03 eV (0.7 kcal/mol) higher in energy relative to the *C*_2v_ minimum, was found to correspond to the transition state for the bowl inversion process. The optimized geometry parameters for NiNc are in excellent agreement with the X-ray structures of several Ni *meso*-diarylnorcorrole derivatives (including CALQIS, CALQOY, CALQUE^12^; YAFSAC, YAFSEF^13^; REMGOI^14^; Table 1). These structures are either planar or slightly domed, consistent with a soft doming potential, and exhibit minimal bond length alternations, in particular minimal differences (<0.02 Å) between adjacent C_*meso*_-C_*α*_ bonds. The X-ray structures of certain other NiNc derivatives (YEQKUC^6^; CALRAL^12^; MUJTIW, MUJTOC^15^, on the other hand, exhibit larger differences (>0.04 Å) between adjacent C_*meso*_-C_*α*_ bonds. To this list may be added a slightly saddled CuNc (YEHTOX)^11^ and a strongly domed PdNc (YEHTEN)^11^ structure, which exhibit an intermediate difference (~0.03 Å) between adjacent C_*meso*_-C_*α*_ bonds. Significant bond localization has been observed for several NiNc derivatives with strongly conjugating *β*-substituents such as cyano^15^, nitro^16^ or amino^17^; these systems are not within the purview of the present study. The overall body of results strongly suggests that although NiNc has a symmetric, delocalized global minimum, bond alternation does correspond to a soft distortion and may manifest itself for certain substitution patterns and crystal environments.

**Table 1:** Selected crystallographic geometry parameters (Å) for relevant norcorrole derivatives.

Refcode	Compound	d(M-N)_mean_^a^	d(N-N)_||_^b^	d(N-N)_⊥_^c^	D(C_α_-C_m_)_mean_^d^	D(C_α_-N)_mean_^e^	geometry	Metal	Ref
YEHTEN	Palladium *meso*,*meso’*-dimesitylnorcorrole	1.915	2.533	2.837	0.035	0.011	domed	Pd	^11^
YEHTOX	Copper *meso*,*meso’*-dimesitylnorcorrole	1.789	2.427	2.628	0.019	0.007	slightly saddled	Cu	^11^
CALQIS	Nickel *meso*-(4-dimethylaminophenyl)-*meso*’-phenylnorcorrole	1.783	2.419	2.618	0.007	0.004	slightly domed	Ni	^12^
CALQOY	Nickel *meso*-(4-dimethylaminophenyl)-*meso*’-phenylnorcorrole	1.782	2.427	2.610	0.003	0.003	planar	Ni	^12^
CALQUE	Nickel *meso*-(4-cyanophenyl)-*meso*’-(4-dimethylaminophenyl)norcorrole	1.771	2.423	2.583	0.000	0.000	planar	Ni	^12^
CALRAL	Nickel *meso*-[3,5-bis(trifluoromethyl)phenyl]-*meso*’-(4-dimethylaminophenyl)norcorrole	1.783	2.422	2.610	0.049	0.018	domed	Ni	^12^
CALRAL	Nickel *meso*-[3,5-bis(trifluoromethyl)phenyl]-*meso*’-(4-dimethylaminophenyl)norcorrole	1.780	2.422	2.611	0.033	0.009	planar	Ni	^12^
CALREP	Nickel *meso*-(4-dimethylaminophenyl)-*meso*’-pentafluorophenylnorcorrole, dichloromethane solvate	1.786	2.425	2.624	0.006	0.021	planar	Ni	^12^
YAFSAC	Nickel *meso*,*meso*’-diphenylnorcorrole	1.789	2.424	2.614	0.019	0.008	domed	Ni	^13^
YAFSAC	Nickel *meso*,*meso*’-diphenylnorcorrole	1.779	2.419	2.609	0.019	0.013	slightly waved	Ni	^13^
YEKQUC	Nickel *meso*,*meso’*-dimesitylnorcorrole	1.779	2.406	2.622	0.044	0.015	planar	Ni	^6^
REMGOI	Nickel 3-(4-dimethylaminophenyl)-5,14-dimesitylnorcorrole), dichloromethane solvate	1.783	2.415	2.621	0.006	0.008	slightly domed	Ni	^14^

^a^average M-N distance; ^b^N…N distances parallel to direct pyrrole-pyrrole bonds; ^c^N…N distances perpendicular to direct pyrrole-pyrrole bonds; ^d^difference in length between adjacent C_α_-C_meso_ bonds, averaged over the whole molecule; ^e^difference in length between adjacent C_α_-N bonds, averaged over the whole molecule.

**Figure 3:**
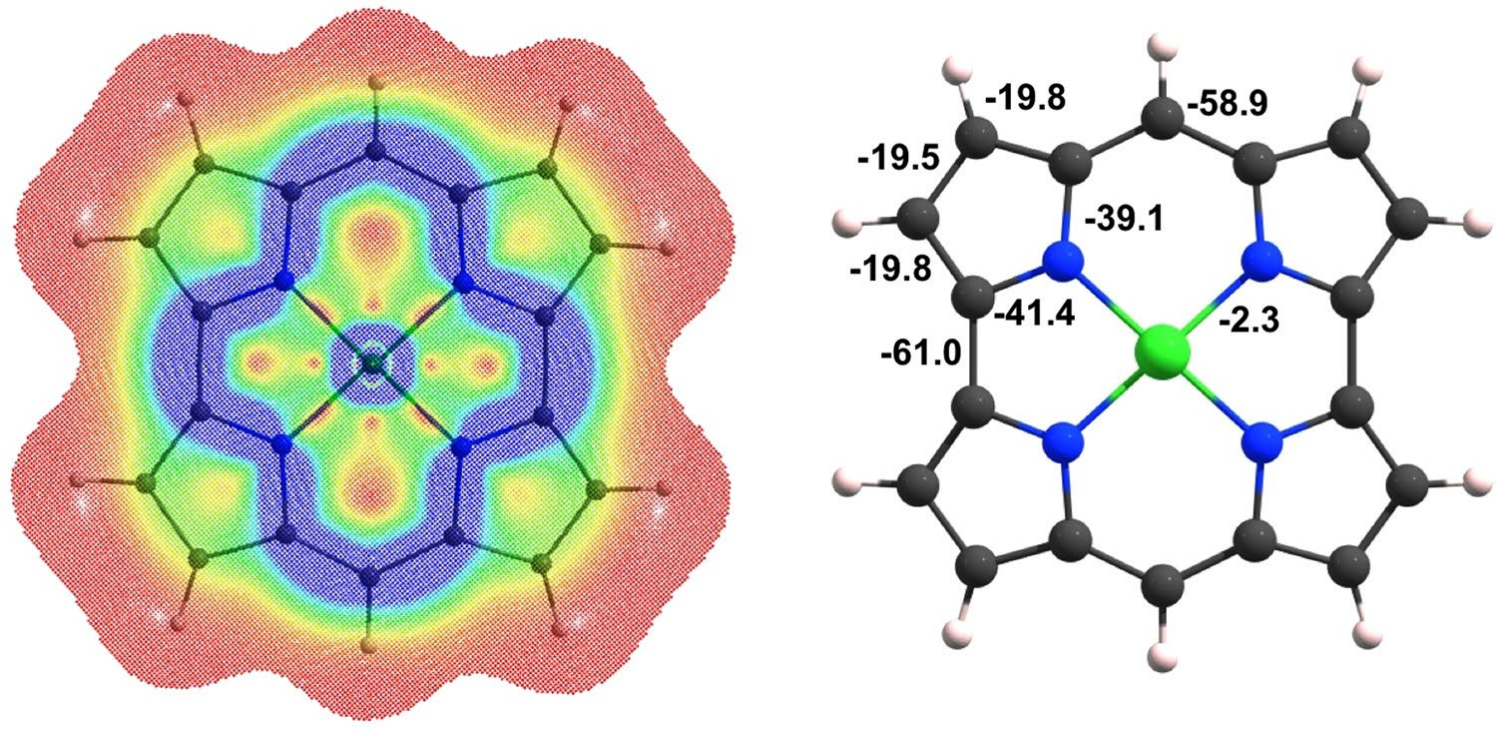
Left: Current density profile of NiNc for a magnetic field applied along the C_2_ axis of symmetry depicted 1 bohr above the ring plane. Red to blue colors represents weak (0.0 au) to strong (0.001 au) current densities. Right: Integrated current intensities (nA/T).

To investigate the question of magnetic antiaromaticity of NiNc, we visualized the magnetically induced current density profile (as previously done for a variety of porphyrin, hydroporphyrin^18^, carbaporphyrin^19^, corrole, and isocorrole^20^ derivatives) and also calculated the bond magnetizabilities^21,22^ at the B3LYP/def2-TZVP^23^ level (Fig. 3). Furthermore, we decomposed both the current intensities and the bond magnetizabilities into σ and π components (Table 2). According to the quantum theory of atoms in molecules (QTAIM)^24^, the total magnetizability of a closed-shell molecule can be decomposed into atomic and bond magnetizabilities, with the latter providing an indirect measure of the total current density flux through the interatomic surface between two neighboring atoms^25^. Keith and Bader showed that, unlike for aliphatic chains, the atomic and bond magnetizabilities of benzene are significantly anisotropic; the out-of-plane components of the magnetizabilities were found to be about three times larger than the in-plane components^26,27^. In a series of papers, one of us demonstrated that the out-of-plane bond magnetizability provides a safe index for assessing aromaticity even in complicated cases^21,22,28,29^, where simple probes such as nucleus independent chemical shift (NICS) and its variants fail^30^. Table 2 shows that the π-framework of NiNc sustains a strong paratropic electronic current (which is associated with positive bond magnetizabilities) that offsets a much smaller diatropic current along the σ–framework. The strong paratropic current is a clear indication of magnetic antiaromaticity of NiNc.

**Table 2:** Out-of-plane bond magnetizabilities (${{\boldsymbol{\chi }}}_{{\boldsymbol{b}}}^{{\boldsymbol{zz}}}$, cgs-ppm) and magnetically induced current intensities (MICI, nA/T) for NiNc. See diagram in the leftmost column for definition of bonds a-h.

			a	b	c	d	e	f	g	h
	${{\boldsymbol{\chi }}}_{{\boldsymbol{b}}}^{{\boldsymbol{zz}}}$	Total	+23.4	+5.1	+8.6	+5.4	+22.2	+9.3	+15.5	+20.7
		σ	−6.8	−4.8	−3.1	−4.7	−5.7	−3.9	−5.2	−5.7
		π	+30.2	+9.9	+11.7	+10.1	+27.9	+13.2	+20.7	+26.4
	MICI	Total	−61.0	−19.8	−19.5	−19.8	−58.9	−39.1	−41.4	−2.3
		σ	+6.3	+3.6	+11.8	+4.5	−0.4	+2.3	+4.8	+0.3
		π	−67.3	−23.4	−31.3	−24.3	−58.5	−42.4	−46.2	−2.6

**Figure 4:**
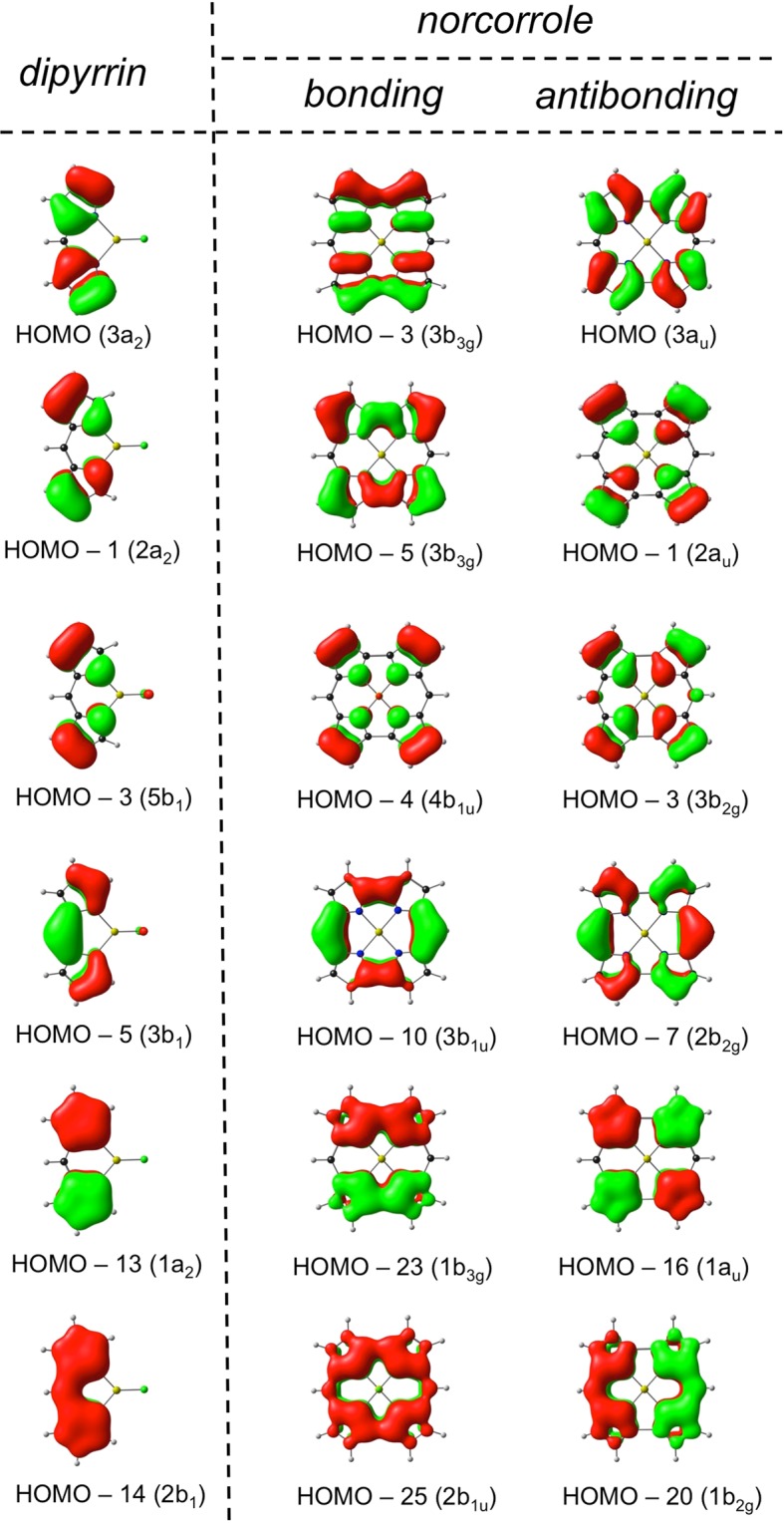
Fragment MO analysis of MgNc in terms of those of Mg(dipy)F.

As noted above, nothing in chemical theory actually rules out a delocalized structure for an antiaromatic system. Whether bond localization will occur in a given case depends on the relative importance of π and σ distortivities of the system^31–33^. Typically, for antiaromatic systems, the former wins out^2–4^. The exceptional nature of NiNc in this regard is perhaps best appreciated in terms of the great stability of the two dipyrrin anion (dipy) fragments that make up the molecule. Such an interpretation is fully in line with a fragment molcular orbital (MO) analysis, which we carried out for the planar *D*_2h_ complex MgNc. As shown in Fig. 4, all 12 occupied π MOs of MgNc may be regarded as bonding and antibonding combinations of the 6 occupied π MOs of the Mg(dipy)F fragments. This statement is actually not a trivial one, the key implication being that none of the occupied π MOs of MgNc owes its origin to any of the *unoccupied* π MOs of the Mg(dipy)F fragments. The two dipyrrin halves thus largely maintain their electronic integrity as part of the norcorrole macrocycle. Notably, such an interpretation is consistent with elementary notions of organic functional groups: as vinylogous amidinates, dipyrrin anions are indeed expected to resist structural distortions such as double bond localization.

## Conclusion

Our knowledge of antiaromatic porphyrinoid systems has deepened greatly in recent years. Thus, a variety of spectroscopic features have now been recognized as hallmarks of such systems^34^. As low-bandgap materials, antiaromatic porphyrinoids in general and norcorrole derivatives in particular are potentially of great interest as components of molecular electronic circuits^35^. Against this exciting backdrop, we have confirmed that simple norcorrole derivatives afford unique examples of symmetric, delocalized, antiaromatic systems. Fragment MO analysis suggests that these seemingly contradictory attributes reflect the great stability of the two dipyrrin halves of the molecule. In other words, the energetic imperative of delocalized bonding within the dipyrrin fragments overrules that of antiaromaticity-related bond length alternation.

## Methods

Geometry optimization studies and the fragment MO analysis were carried out with the ADF2017^36,37^ program system using methods described above. All optimized structures (see Supplementary information for coordinates) were confirmed as local minima via frequency analyses. To obtain current density plots and current intensities, geometry optimizations and GIAO NMR calculations were performed at the B3LYP/def2-TZVP level with Gaussian 09 rev. D1^38^. The NMR computations were further analyzed with the AIMAll (version 16.05.18) suite of programs^39^. Current densities were obtained within the context of quantum theory of atoms in molecules as developed by Keith and Bader^40–44^.

## Electronic supplementary material

Supplementary Information
