# Supplementary material for: Norcorrole as a Delocalized, Antiaromatic System
Source: Sci Rep. 2019 Mar 19;9:4852. doi: 10.1038/s41598-019-39972-y (PMC6425022; doi:10.1038/s41598-019-39972-y)
Supplement: Supplementary file 1 — Supplementary Information [file 41598_2019_39972_MOESM1_ESM.pdf]

Supporting information

Norcorrole As an Example of A Delocalized, Antiaromatic System

Jeanet Conradie,* Cina Foroutan-Nejad* and Abhik Ghosh*

Symmetrized BLYP-D3/TZ2P Cartesian coordinates (Å)

Table of Contents

1. H_2Nc (C_i) 2
2. NiNc (C_{2V}) 2
3. NiDBNc (C_{2v}) 3
4. NiTBNc (D_{2h}) 4

1. H_2Nc (C_i)

C	0.559682000	-3.762876000	-0.151823000
C	0.842799000	3.835846000	0.240017000
C	0.889001000	-2.445179000	0.211427000
C	1.361614000	2.560056000	-0.073401000
C	2.048260000	-1.524366000	0.212510000
C	2.625252000	1.918210000	0.127302000
C	2.800927000	0.542551000	0.139111000
C	3.455184000	-1.589045000	0.552408000
C	3.920801000	-0.299102000	0.508252000
C	-0.559682000	3.762876000	0.151823000
C	-0.842799000	-3.835846000	-0.240017000
C	-0.889001000	2.445179000	-0.211427000
C	-1.361614000	-2.560056000	0.073401000
C	-2.048260000	1.524366000	-0.212510000
C	-2.625252000	-1.918210000	-0.127302000
C	-2.800927000	-0.542551000	-0.139111000
C	-3.455184000	1.589045000	-0.552408000
C	-3.920801000	0.299102000	-0.508252000
H	0.389429000	0.793837000	-0.509587000
H	1.255840000	-4.552808000	-0.377565000
H	1.418435000	4.696177000	0.540775000
H	3.467720000	2.549074000	0.381683000
H	4.010046000	-2.471462000	0.827878000
H	4.921692000	0.034042000	0.734991000
H	-0.389429000	-0.793837000	0.509587000
H	-1.255840000	4.552808000	0.377565000
H	-1.418435000	-4.696177000	-0.540775000
H	-3.467720000	-2.549074000	-0.381683000
H	-4.010046000	2.471462000	-0.827878000
H	-4.921692000	-0.034042000	-0.734991000
N	0.276835000	1.802895000	-0.404277000
N	1.710795000	-0.285950000	-0.063597000
N	-0.276835000	-1.802895000	0.404277000
N	-1.710795000	0.285950000	0.063597000

2. NiNc (C_{2v})

C	0.000000000	3.200711000	-0.078438000
C	0.000000000	-3.200711000	-0.078438000
C	1.256168000	2.563542000	0.009514000
C	1.256168000	-2.563542000	0.009514000
C	2.566530000	0.746749000	0.025937000
C	2.566530000	-0.746749000	0.025937000
C	2.588977000	2.981463000	-0.311045000
C	2.588977000	-2.981463000	-0.311045000
C	3.400830000	1.851761000	-0.306773000

C	3.400830000	-1.851761000	-0.306773000
C	-1.256168000	2.563542000	0.009514000
C	-1.256168000	-2.563542000	0.009514000
C	-2.566530000	0.746749000	0.025937000
C	-2.566530000	-0.746749000	0.025937000
C	-2.588977000	2.981463000	-0.311045000
C	-2.588977000	-2.981463000	-0.311045000
C	-3.400830000	1.851761000	-0.306773000
C	-3.400830000	-1.851761000	-0.306773000
H	0.000000000	4.255761000	-0.323446000
H	0.000000000	-4.255761000	-0.323446000
H	2.898850000	3.989407000	-0.536678000
H	2.898850000	-3.989407000	-0.536678000
H	4.453784000	1.818049000	-0.531945000
H	4.453784000	-1.818049000	-0.531945000
H	-2.898850000	3.989407000	-0.536678000
H	-2.898850000	-3.989407000	-0.536678000
H	-4.453784000	1.818049000	-0.531945000
H	-4.453784000	-1.818049000	-0.531945000
N	1.334340000	1.217871000	0.242969000
N	1.334340000	-1.217871000	0.242969000
N	-1.334340000	1.217871000	0.242969000
N	-1.334340000	-1.217871000	0.242969000
Ni	0.000000000	0.000000000	0.365594000

3. NiDBNc (C_{2v})

C	0.000000000	0.000000000	4.023968000
C	0.000000000	0.000000000	-2.415645000
C	0.000000000	1.256938000	3.377205000
C	0.000000000	1.258517000	-1.779111000
C	0.000000000	2.578061000	1.559471000
C	0.000000000	2.585664000	0.065244000
C	0.000000000	2.619023000	3.823865000
C	0.000000000	2.630965000	-2.235667000
C	0.000000000	3.219020000	-3.500107000
C	0.000000000	3.438633000	2.697004000
C	0.000000000	3.470183000	-1.060776000
C	0.000000000	4.600270000	-3.597884000
C	0.000000000	4.862095000	-1.185832000
C	0.000000000	5.415872000	-2.452413000
C	0.000000000	-1.256938000	3.377205000
C	0.000000000	-1.258517000	-1.779111000
C	0.000000000	-2.578061000	1.559471000
C	0.000000000	-2.585664000	0.065244000
C	0.000000000	-2.619023000	3.823865000
C	0.000000000	-2.630965000	-2.235667000
C	0.000000000	-3.219020000	-3.500107000
C	0.000000000	-3.438633000	2.697004000
C	0.000000000	-3.470183000	-1.060776000

C	0.000000000	-4.600270000	-3.597884000
C	0.000000000	-4.862095000	-1.185832000
C	0.000000000	-5.415872000	-2.452413000
H	0.000000000	0.000000000	5.107167000
H	0.000000000	0.000000000	-3.498805000
H	0.000000000	2.607918000	-4.393955000
H	0.000000000	2.949079000	4.850423000
H	0.000000000	4.515758000	2.689949000
H	0.000000000	5.064904000	-4.575334000
H	0.000000000	5.494121000	-0.307153000
H	0.000000000	6.491504000	-2.568619000
H	0.000000000	-2.607918000	-4.393955000
H	0.000000000	-2.949079000	4.850423000
H	0.000000000	-4.515758000	2.689949000
H	0.000000000	-5.064904000	-4.575334000
H	0.000000000	-5.494121000	-0.307153000
H	0.000000000	-6.491504000	-2.568619000
N	0.000000000	1.322916000	2.012740000
N	0.000000000	1.341663000	-0.414420000
N	0.000000000	-1.322916000	2.012740000
N	0.000000000	-1.341663000	-0.414420000
Ni	0.000000000	0.000000000	0.792767000

4. **NiTBNc (*D_{2h}*)**

C	0.000000000	0.000000000	3.221326000
C	0.000000000	0.000000000	-3.221326000
C	0.000000000	1.239464000	2.537698000
C	0.000000000	1.239464000	-2.537698000
C	0.000000000	2.616050000	0.692493000
C	0.000000000	2.616050000	-0.692493000
C	0.000000000	2.641545000	3.008616000
C	0.000000000	2.641545000	-3.008616000
C	0.000000000	3.183666000	4.279735000
C	0.000000000	3.183666000	-4.279735000
C	0.000000000	3.487408000	1.857835000
C	0.000000000	3.487408000	-1.857835000
C	0.000000000	4.571718000	4.420104000
C	0.000000000	4.571718000	-4.420104000
C	0.000000000	4.865416000	2.012438000
C	0.000000000	4.865416000	-2.012438000
C	0.000000000	5.398617000	3.300171000
C	0.000000000	5.398617000	-3.300171000
C	0.000000000	-1.239464000	2.537698000
C	0.000000000	-1.239464000	-2.537698000
C	0.000000000	-2.616050000	0.692493000
C	0.000000000	-2.616050000	-0.692493000
C	0.000000000	-2.641545000	3.008616000
C	0.000000000	-2.641545000	-3.008616000
C	0.000000000	-3.183666000	4.279735000
C	0.000000000	-3.183666000	-4.279735000

C	0.000000000	-3.487408000	1.857835000
C	0.000000000	-3.487408000	-1.857835000
C	0.000000000	-4.571718000	4.420104000
C	0.000000000	-4.571718000	-4.420104000
C	0.000000000	-4.865416000	2.012438000
C	0.000000000	-4.865416000	-2.012438000
C	0.000000000	-5.398617000	3.300171000
C	0.000000000	-5.398617000	-3.300171000
H	0.000000000	0.000000000	4.300586000
H	0.000000000	0.000000000	-4.300586000
H	0.000000000	2.543791000	5.152568000
H	0.000000000	2.543791000	-5.152568000
H	0.000000000	5.010286000	5.409116000
H	0.000000000	5.010286000	-5.409116000
H	0.000000000	5.520466000	1.152226000
H	0.000000000	5.520466000	-1.152226000
H	0.000000000	6.472876000	3.430606000
H	0.000000000	6.472876000	-3.430606000
H	0.000000000	-2.543791000	5.152568000
H	0.000000000	-2.543791000	-5.152568000
H	0.000000000	-5.010286000	5.409116000
H	0.000000000	-5.010286000	-5.409116000
H	0.000000000	-5.520466000	1.152226000
H	0.000000000	-5.520466000	-1.152226000
H	0.000000000	-6.472876000	3.430606000
H	0.000000000	-6.472876000	-3.430606000
N	0.000000000	1.315470000	1.207744000
N	0.000000000	1.315470000	-1.207744000
N	0.000000000	-1.315470000	1.207744000
N	0.000000000	-1.315470000	-1.207744000
Ni	0.000000000	0.000000000	0.000000000
